# Poster Session I - A60 A SURVEY EVALUATING USER PERCEPTIONS OF A COMMERCIAL ARTIFICIAL INTELLIGENCE-BASED POLYP DETECTION SYSTEM DURING COLONOSCOPY

**DOI:** 10.1093/jcag/gwaf042.060

**Published:** 2026-02-13

**Authors:** J Singh, L Hookey

**Affiliations:** Queen’s University Faculty of Health Sciences, Kingston, ON, Canada; Kingston Health Sciences Centre, Kingston, ON, Canada

## Abstract

**Background:**

Colorectal cancer is a largely preventable disease that can be prevented through detection and removal of precancerous polyps (adenomas), most commonly through colonoscopy. However, despite its effectiveness, finding all polyps remains a challenge, with several adjuncts being proven to assist in polyp detection. Artificial intelligence (AI) systems, such as FUJIFILM’s CAD EYE, have been developed to support real-time polyp detection during colonoscopy treatments. While clinical trials suggest potential benefits, the success of such tools relies primarily on clinician perceptions and usability in routine practice.

**Aims:**

This cross-sectional study aimed to assess healthcare professionals’ experiences and perceptions of CAD EYE, with a focus on its acceptance, perceived effectiveness, and its impact on workflow during colonoscopy.

**Methods:**

Nineteen healthcare professionals, including gastroenterologists, fellows, and trainees, participated in a structured and confidential survey. Survey responses were analyzed using descriptive statistics and thematic analysis. One participant was excluded due to lack of experience with the software.

**Results:**

Of the 18 surveyed participants, 4 reported regular use of CAD EYE, 5 reported no present use, and 9 reported occasional use. Compared with their past usage, 5 participants had completely stopped using CAD EYE, 5 reported reduced use, and 6 reported increased use. Yet, on a 5-point scale (5 = very effective), CAD EYE was rated an average of 2.4. In terms of workflow, 11 participants indicated that CAD EYE made their work slower, 6 reported it made no difference, and 1 found it faster.

Reasons for use or non-use fell into eight distinct categories: distraction (n = 6), negative workflow impact (n = 3), limited experience (n = 1), ineffective for polyp detection (n = 3), beneficial for polyp detection (n = 3), false positives (n = 5), second-opinion support (n = 4), and noise annoyance (n = 4). CAD EYE was most frequently used during general polyp detection on withdrawal (n = 4), polyp characterization (n = 8), and procedures involving high-risk or special populations (n = 1).

Perceived benefits included improved polyp detection (n = 6), whereas limitations included workflow disruption from visual and audio cues (n = 6), oversensitivity and false positives (n = 7), and a general need for improvement (n = 2).

**Conclusions:**

Although potential clinical benefits in polyp detection, CAD EYE’s acceptance in the one center’s clinical group is limited by perceived inefficiencies and frequent false-positive results. With enhanced specificity and customizable alerts, CAD EYE could significantly improve user experience and its implementation in the field.

**Funding Agencies:**

None

